# Investigation of market herbal products regulated under different categories: How can HPTLC help to detect quality problems?

**DOI:** 10.3389/fphar.2022.925298

**Published:** 2022-08-08

**Authors:** Débora A. Frommenwiler, Eike Reich, Maged H. M. Sharaf, Salvador Cañigueral, Christopher J. Etheridge

**Affiliations:** ^1^ CAMAG Laboratory, Muttenz, Switzerland; ^2^ Unit of Pharmacology, Pharmacognosy and Therapeutics, Faculty of Pharmacy and Food Sciences, Universitat de Barcelona, Barcelona, Spain; ^3^ HPTLC Association, Rheinfelden, Switzerland; ^4^ British Herbal Medicine Association (BHMA), Exeter, United Kingdom

**Keywords:** HPTLC, coneflower, milk thistle (Silybum marianum), black cohosh, food supplement, THMP

## Abstract

**Background:** Herbal products regulated under different categories were found to be of different quality. This has been demonstrated by the increasing number of reports on the quality of herbal products in the scientific literature. Proper identification is an effective way to address this concerning issue early on in a products’ manufacturing process.

**Objectives:** To assess the quality of milk thistle, coneflower and black cohosh herbal drugs, preparations and products commercialized under different regulatory categories, and to illustrate the usefulness of HPTLC as a tool for evaluating quality.

**Methods:** HPTLC methods were adapted from the European Pharmacopeia’s monographs for milk thistle fruits, black cohosh and purple coneflower. Additional detection modes beyond those described in the monographs were employed, and the entire HPTLC fingerprints were used for examination of identity and purity of the investigated samples.

**Results:** All products regulated as Traditional Herbal Medicinal Products were shown to be of high quality: their fingerprints were consistent and without unexpected zones. A significant number of food supplements show quality issues (mainly adulterations): 52.4% of milk thistle, 33.3% of coneflower, and 45.5% of black cohosh products. The same was observed in 66.6% of black cohosh herbal drugs and preparations.

## 1 Introduction

Herbal products containing the same ingredient and regulated under different categories can be of differing quality, because different regulatory evaluations do not always require the same scientific scrutiny. The two main categories for marketing herbal products are licensed medicines (herbal medicinal products and traditional herbal medicinal products) and food supplements. A company can decide in which regulatory framework it wants to market a product, but the former has higher regulatory constraints and higher costs than the latter. Although companies producing food supplements are obliged to comply with good manufacturing practices (GMP), in many countries, those products do not undergo pre-evaluation/approval by a national authority. According to Low et al. (2017), mandatory pre-marketing evaluation of products regulated under non-drug categories can increase the burden on both regulators and business, and thus seem to be an unrealistic solution and a reason that could explain the variation in quality of herbal products in different markets. The cost of analysis and use of expensive equipment for analysis can further exacerbate this burden.

Quality control of herbal products starts with proper identification of their herbal ingredients. By using the right set of tools, additional quality parameters (beyond establishing the correct identity) can be assessed within the same analysis (e.g. purity of the material). Techniques such as HPTLC, recommended by pharmacopoeias to evaluate the identity of herbals, can deliver valuable supplementary data without the need for additional analyses. In addition, multiple samples can be tested at the same time and under the same exact conditions on one plate. With proper methodology, multiple samples can be compared across HPTLC plates that have been developed at different times and/or in different laboratories. In addition, the entire fingerprint of a sample, sometimes in multiple detection modes, can be utilized for assessment instead of just looking at only a few zones as described in the acceptance criteria of typical monographs. Such extended evaluation of a single HPTLC analysis, in comparison to other tools, is useful for detecting zones that may indicate quality problems (Cañigueral et al., 2018).

To illustrate this concept, case studies have been conducted on milk thistle, coneflower, and black cohosh. The first two case studies were carried out in collaboration with the British Herbal Medicine Association (BHMA). Herbal ingredients were chosen based on their market importance. In 2016, they were listed among the top-20 selling products in the United States mainstream market (Smith et al., 2017). They are also well-known ingredients in the European market.

Milk thistle, the dried fruit of *Silybum marianum* (L.) Gaertn., is one of the most frequently sold herbal products for treatment and relief of dyspepsia and digestive complaints of hepatic origin. Its preparation is usually standardized to contain 70–80% of three flavonolignans (silybin, silychristin, and silydianin), collectively known as silymarin. A high concentration of flavonolignans in milk thistle extract is recommended because of their poor absorption in the gastrointestinal tract (Blumenthal et al., 2000). Therefore, many products in the market declare to be standardized to contain high levels of silymarin. Another major constituent of milk thistle is the flavonoid taxifolin, which has also been associated with inhibitory activity against liver disease (Das et al., 2021). The main reported adulterations of milk thistle are depleted milk thistle extracts, *Silybum eburneum* Coss. and Dur., and synthetic colorants (McCutcheon, 2020). Milk thistle is recognized as a Traditional Herbal Medicinal Product (THMP) in the European Union and in a similar category known as Traditional Herbal Registration medicinal product (THR) in the United Kingdom (Committee on Herbal Medicinal Products European Medicines Agency, 2018b). However, it is also widely sold as a food supplement.

The term coneflower refers to several *Echinacea* species. In particular, aerial parts and/or roots of three species are used medicinally: *E. purpurea* (L.) Moench (purple coneflower), *E. angustifolia* DC. (narrow-leaf coneflower) and *E. pallida* (Nutt.) Nutt (pale coneflower). The roots of the three species and aerial parts of E. purpurea are used mainly for preventing and treating common cold. The roots of *E. purpurea* are also used for relief of spots and pimples due to mild acne, and the aerial parts for treatment of small superficial wounds. Proposed active constituent groups of these coneflowers include polysaccharides, glycoproteins, caffeic acid derivatives, and alkylamides (Ardjomand-Woelkart and Bauer, 2015). In the European Union, preparations of roots of the three species are recognized as THMP and those from fresh aerial parts of purple coneflower are accepted as both, THMP and Well Established Use (WEU) medicinal products (Blumenthal et al., 2000; Committee on Herbal Medicinal Products European Medicines Agency, 2012; Committee on Herbal Medicinal Products European Medicines Agency, 2015; Committee on Herbal Medicinal Products European Medicines Agency, 2017b; Committee on Herbal Medicinal Products European Medicines Agency, 2018a). In the UK, medicinal products containing coneflower preparations are sold as THR products. In both, the European Union and the United Kingdom, coneflower products are also sold as food supplements. They often contain one or more *Echinacea* sp. or different parts of the same or different species.

Poor quality of coneflower products has been reported for a long time. At the end of the last century, coneflower products in the United States market often contained *Parthenium integrifolium* L. as a substitution, which is no longer the case (Hobbs, 1994). Species mix-up can also happen between members of the *Echinacea* genus. According to Ardjomand-Woelkart and Bauer (Ardjomand-Woelkart and Bauer, 2015), roots of *E. angustifolia* and *E. pallida* are often confused due to their morphological similarities. The former species is endangered in the wild. Nowadays, coneflowers products mostly contain *E. purpurea* root. Spelman stated that confusion of species is still almost certainly occurring when researchers and companies do not have proper identification procedures in place (Spelman, 2013).

Preparations of black cohosh, the dried root and rhizome of *Actaea racemosa* L., are widely used in the United States, Canada, Europe, Australia, and elsewhere, principally for treatment of menopausal symptoms. The two main compound classes of this herbal drug are triterpene glycosides and polyphenolic derivatives (European Scientific Cooperative on Phytotherapy E/S/C/O/P, 2011).

For economic reasons, black cohosh products in North America are known to be adulterated with related species from China. The most common of those are *Actaea cimicifuga* L. and *Actaea dahurica* (Turcz. ex Fisch. and C.A.Mey.) Franch. Intentional adulteration happens mainly because of the cheaper price of Chinese powdered material and extract, which may be as low as one-quarter of that of the authentic black cohosh. Accidental adulteration with Chinese material can also happen because of confusion in nomenclature. For example, Chinese species of *Actaea* and *Serratula chinensis* S. Moore are sold under the name black cohosh through internet shops. Admixture with American species (e.g., *A. podocarpa* DC.*, A. pachypoda* Elliott, *A. rubra* (Aiton) Willd., and *A. cordifolia* DC.) also occurs because they share the same habitat and closely resemble black cohosh (Foster, 2013) when the underground parts are harvested in the fall. In the European Union, black cohosh products are sold as WEU medicinal products (Committee on Herbal Medicinal Products European Medicines Agency, 2017a) and as THR in the United Kingdom, or as food supplements, while in the United States, they are considered dietary supplements.

The objective of the case studies was to evaluate the quality of different herbals as a function of their regulatory category and to show the usefulness of the HPTLC fingerprint as a tool for detecting quality issues, particularly adulterations. Samples were evaluated with the HPTLC methods of the repective Ph. Eur. monographs with some modifications. Interpretation was based on the entire fingerprint, exceeding the table description of the Ph. Eur., and additional detection modes.

## 2 Materials and methods

### 2.1 Chemical reference standards, reagents, and apparatus

The chemical reference standards silybin (98% pure) and caffeic acid (98% pure) were obtained from Sigma Aldrich (St. Louis, United States), silydianin, chlorogenic acid (97% pure), caftaric acid (90% pure), and chicoric acid (97% pure) from USP (Rockville, United States), and silychristin (97.9% pure), dodec-2-ene-8,10-diynoic acid isobutylamide, and isoferulic acid (97% pure) from Chromadex (Los Angeles, United States). Taxifolin (85% pure) was purchased from Extrasynthese (Genay, France), and β-sitosterol (95% pure), ursolic acid (97% pure), echinacoside (95% pure), dodeca-2E,4E,8Z, 10*E/Z*-tetraenoic acid isobutylamide (93% pure), cynarin (96% pure), actein (95% pure), and cimifugin (97% pure) from Phytolab (Vestenbergsgreuth, Germany).

Solvents (≥95% pure) and reagents were purchased from Roth (Karlsruhe, Germany), Acros (Gent, Belgium), Fisher Scientific (Hampton, United States), and Merck (Darmstadt, Germany). Silica gel 60 F_254_ HPTLC glass plates (20 × 10 cm) were obtained from Merck (Darmstadt, Germany).

HPTLC instruments from CAMAG (Muttenz, Switzerland) were used, including Automatic TLC Sampler (ATS 4), Automatic Development Chamber (ADC 2) with humidity control, Plate Heater 3, TLC Visualizer 2, and Immersion Device 3.

### 2.2 Samples

Thirty-one products of milk thistle and twenty-three products of coneflower were acquired from the internet, local shops, and pharmacies in the United Kingdom, as part of the collaboration with the British Herbal Medicine Association. They included tablets, chewable tablets, capsules, tinctures, and liquid extracts, sold as food supplements or traditional herbal medicinal products. Their labels claimed contents of either standardized extract, extract, a mixture of extract and herbal drug, or dried herbal drug.

Sixty samples of products, herbal drugs and herbal preparations (e.g., extracts) labeled as black cohosh, including tea bags, capsules, tablets of plant material and/or extracts, and herbal ingredients (powdered herbal drug and extracts) were acquired from the internet, and the market in the United States by one of the coauthors (MHMS).

A list of samples and their specifications is presented in the supplementary information (Supplementary Tables S1–3).

### 2.3 Preparation of test solutions

#### 2.3.1 Milk thistle and coneflower products

Products were extracted with methanol to contain the equivalent of 100 mg of dried herbal drug, dried or liquid extract per mL of solution. If the drug extract ratio of extracts was declared, it was used to calculate the equivalent amount of herbal drug used in the preparation. The mixtures were sonicated for 10 min, centrifuged, and the supernatants used as test solutions. For analysis of alkylamides in coneflower products, the test solutions were prepared in dichloromethane following the same procedure.

Milk thistle tinctures (MT20 and MT14), which did not declare the drug extract ratio were directly applied onto the plate. Samples MT12, 13 and 30 were prepared at 5 mg/ml, MT8 at 25 mg/ml and ECH16 at 20 mg/ml. These concentrations were adopted due to the overloaded fingerprints or matrix disturbance, observed in these samples during initial experiments.

#### 2.3.2 Black cohosh products, herbal drugs and preparations

Products were extracted with ethanol and water (50:50 v/v) to contain the equivalent of 50 mg of herbal drug, extract, or combined herbal drug and extract per mL of solution. Mixtures were sonicated for 10 min, centrifuged, and the supernatants were used as test solutions.

### 2.4 HPTLC parameters

HPTLC was performed with general parameters specified in Ph. Eur. 2.8.25.

#### 2.4.1 Milk thistle products

The HPTLC method was adapted from the Ph. Eur. monograph for milk thistle fruits (European Directorate for the Quality of Medicines and Healthcare, 2020a) as published by the HPTLC Association (HPTLC Association, 2019) and adopted in the United States Pharmacopeia (USP) Dietary Supplements Compendium (DSC) 2019 (United States Pharmacopeial Convention, 2020). Specific parameters are described in Table 1.

**TABLE 1:** HPTLC parameters for identification of milk thistle.

Parameters	Description
Stationary phase	20 × 10 cm glass plates Si 60 F254 (Merck)
SST	0.5 mg/ml of taxifolin, 0.2 mg/ml of silybin, 0.1 mg/ml of silychristin and silydianin, individually prepared in methanol
Application volume	2 μL of test and References solutions
Developing solvent	Toluene, ethyl formate, formic acid 40:50:5 (v/v/v)
Development	20 min saturation (with filter paper), 10 min conditioning at 33% relative humidity (with MgCl2), 70 mm distance from lower edge, room temperature = 23–27°C
Derivatization reagent 1	Natural Product (NP) reagent: 1 g of diphenylboric acid 2-aminoethyl ester was dissolved in 200 ml of ethyl acetate
Derivatization reagent 2	Polyethylene glycol (PEG) reagent: 10 g of polyethylene glycol (Macrogol) 400 were dissolved in 200 ml of dichloromethane
Derivatization procedure	Plates were heated at 100°C for 5 min and then derivatized by dipping (speed: 3, time: 0) in NP reagent and subsequently in PEG reagent. Plates were heated again for 5 min at 100 °C. Images were taken 1 hour after derivatization
Documentation	White light, UV 254 nm, and UV 366 nm prior to derivatization; UV 366 nm and white light after derivatization

#### 2.4.2 Echinacea products

Two HPTLC methods for the identification of coneflowers roots and aerial parts were adapted from the Ph. Eur. monograph for purple coneflower root (European Directorate for the Quality of Medicines and Healthcare, 2020b) as published by the HPTLC Association, with modifications of sample preparation, application volume, developing distance and derivatization (HPTLC Association, 2014) and used to evaluate coneflower products. The parameters are described in Tables 2, 3. For alkylamides fingerprints, the image after the second heating step was used because it yields stronger zones.

**TABLE 2:** HPTLC parameters for identification of coneflower roots and aerial parts, phenolic compounds fingerprint.

Parameters	Description
Stationary phase	20 × 10 cm glass plates Si 60 F254 (Merck)
SST	0.5 mg/ml of cynarin and echinacoside, 0.1 mg/ml of chlorogenic acid and caffeic acid, individually prepared in methanol
Application volume	2 μL of References solutions and 4 µL of the test solution
Developing solvent	Ethyl acetate, ethyl methyl ketone, water, formic acid 5:3:1:1 (v/v/v/v)
Development	20 min saturation (with filter paper), 10 min conditioning at 33% relative humidity (with MgCl2), 70 mm distance from lower edge, room temperature = 23–27°C
Derivatization reagent 1	NP reagent: 1 g of diphenylboric acid 2-aminoethyl ester was dissolved in 200 ml of ethyl acetate
Derivatization reagent 2	PEG reagent: 10 g of polyethylene glycol (Macrogol) 400 were dissolved in 200 ml of dichloromethane
Derivatization procedure	Plates were heated at 100°C for 3 min and derivatized by dipping (speed: 3, time: 0) into NP reagent and then into PEG reagent
Documentation	White light, UV 254 nm, and UV 366 nm prior to derivatization; UV 366 nm and white light after derivatization

**TABLE 3:** HPTLC parameters for identification of coneflower roots and aerial parts, alkylamides.

Parameters	Description
Stationary phase	20 × 10 cm plates Si 60 F254 (Merck)
SST	0.2 mg/ml ursolic acid, β-sitosterol and dodec-2-ene-8,10-diynoic acid isobutylamide and 0.4 mg/ml of dodeca-2E,4E,8Z,10*E/Z*-tetraenoic acid isobutylamide, individually prepared in methanol
Application volume	2 μL of References solutions and 10 µL of the test solution
Developing solvent	Toluene, ethyl acetate, cyclohexane, formic acid 80:20:10:3 (v/v/v/v)
Development	20 min saturation (with filter paper), 10 min conditioning at 33% relative humidity (with MgCl2), 70 mm distance from lower edge, room temperature = 23–27°C
Derivatization reagent	Anisaldehyde reagent: 20 ml of acetic acid and 10 ml of sulfuric acid was slowly added to 170 ml of ice-cooled methanol and mixed well. The mixture was allowed to cool to room temperature, and then 1 ml of anisaldehyde was added
Derivatization procedure	The plates were dipped (speed: 3, time: 0) into anisaldehyde reagent and then heated 3 min at 100 °C. After documentation, the plates were heated for another 15 min and then documented again
Documentation	White light, UV 254 nm, and UV 366 nm prior to derivatization; UV 366 nm and white light after derivatization and after second heating

#### 2.4.3 Black cohosh products

The HPTLC method for identification of *Cimicifuga racemosa* (syn. *A. racemosa*) of the Ph. Eur. (European Directorate for the Quality of Medicines and Healthcare, 2017b) was used to evaluate black cohosh products, herbal drugs and preparations. The monograph includes three HPTLC methods, which share the same parameters except for the application volume and derivatization reagent. These parameters are summarized in Table 4.

**TABLE 4:** HPTLC parameters for identification of black cohosh.

Parameters	Description
Stationary phase	20 × 10 cm glass plates Si 60 F254 (Merck)
SST	0.1 mg/ml of actein, isoferulic acid and cimifugin are individually prepared in methanol
Application volume	2 μL of References and test solution for identification and 20 µL of test solution for test for the presence of *A. podocarpa*, *A. dahurica*, and *A. cimicifuga*
Developing solvent	Toluene, ethyl formate, formic acid 50:30:20 (v/v/v)
Development	20 min saturation (with filter paper), 10 min conditioning at 0% relative humidity (with molecular sieve), 70 mm distance from lower edge, room temperature = 23–27°C
Derivatization reagent 1 (identification)	Sulfuric acid reagent: 20 ml of sulfuric acid was mixed with 180 ml of methanol. The plate is dipped (time: 0, speed:3) and then heated at 100 °C for 5 min
Derivatization reagent 2 (test for adulteration with *A. dahurica*)	Antimony trichloride reagent: 8 g of Antimony trichloride were mixed with 200 ml of chloroform and shaken until completely dissolved. The plates were dipped (time: 1s, speed: 3) into the solution and then heated at 120 °C for 10 min
Derivatization reagent 3 (test for adulteration with *A. cimicifuga*)	Boric acid, oxalic acid reagent: 4 g of boric acid and 5 g of oxalic acid were individually dissolved in 150 and 50 ml of ethanol absolute, respectively, and then shaken until completely clear. The solutions were combined before derivatization. The plates were dipped (time: 1s, speed: 3) into the solution and heated at 120 °C for 5 min
Documentation	White light, UV 254 nm and UV 366 nm prior to derivatization, UV 366 nm and white light after derivatization

## 3 Results

### 3.1 Milk thistle case study

**FIGURE 1:**
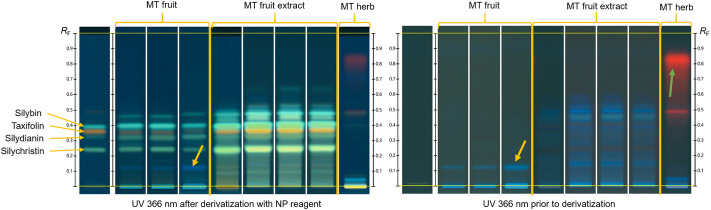
Typical HPTLC fingerprints of milk thistle fruit, fruit extract, and herb prior to (right image) and after derivatization (left image). Left track in each image shows the chromatogram of the reference substances. Yellow arrows: blue zone characteristic of MT fruit, absent in MT fruit extract; Green arrow: chlorophyll zone characteristic of MT herb.

As shown in Figure 1, fingerprints of milk thistle fruit and fruit extract usually show a sequence of four green zones after derivatization, three of which are due to silybin, silydianin, and silychristin. Additionally, an orange zone due to taxifolin is observed just below silybin. Fingerprints of fruit show a blue zone at *R*_F_ 0.13 (yellow arrows) before and after derivatization, which is absent in the extract. Milk thistle herb shows two very faint green zones, one of them at the position of silybin, and, prior to derivatization, an intense red zone due to chlorophylls (green arrow), absent in the fruit and fruit extract.

**FIGURE 2:**
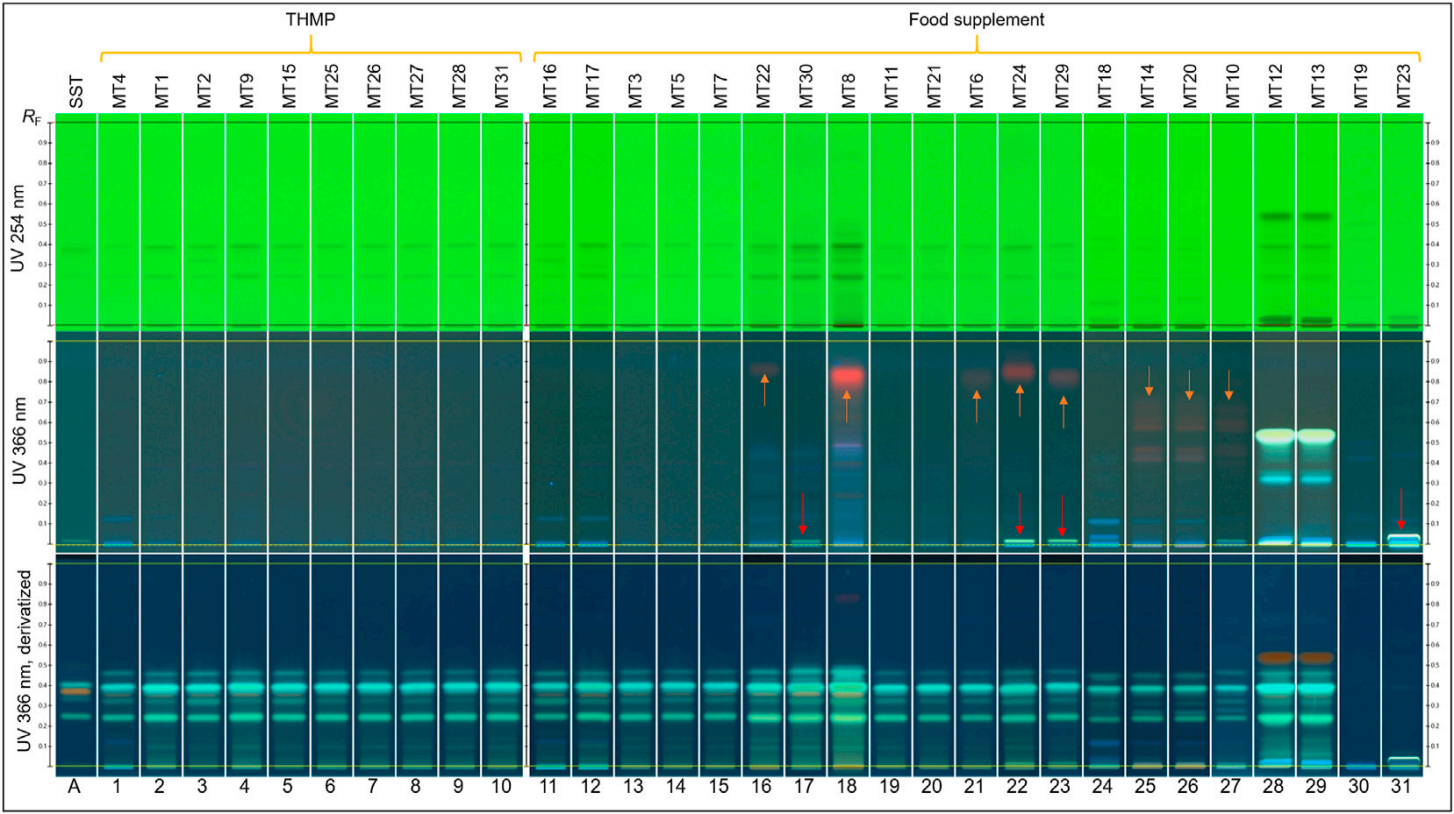
Fingerprints of the 31 milk thistle products in different detection modes, grouped by regulatory categories. Track A: silydianin, silychristin, taxifolin, and silybin in increasing *R*_F_ values. Orange arrows: red zone due to chlorophyll detected in some products; red arrow: an unidentified yellow-white zone detected in some products.

In Figure 2, the milk thistle products are grouped by their regulatory category. Tracks in each category were then arranged based on similarity in visual inspection. All THMP products (tracks 1–10) show homogeneous and consistent fingerprints in regard to the number of zones and their intensities, including those due to flavonolignans and taxifolin. Only one sample declared to contain milk thistle fruit, and as expected, it show a blue zone at *R*_F_ 0.13. Of the twenty-one food supplement products, two declared to contain milk thistle fruit (tracks 11–12) and eight declared to contain MT extracts (tracks 13–18, 28–29). Fingerprints of these ten food supplement products comply with the label information. Two of them (tracks 28–29) show an additional zone at *R*_F_ 0.55, which is likely due to curcuminoids from turmeric extract, which is declared on the product label.

The rest of milk thistle food supplement (11) had questionable fingerprints. Nine of them (tracks 19–27) show the four green zones due to flavonolignans but lacked taxifolin (orange zone). Additionally, some of them have a faint fingerprint under UV 254 nm, in which the zones due to silybin and silychristin are not detected due to their low concentration. Finally, two samples (tracks 30 and 31) lack zones characteristic of fruit or its extracts, which suggest absence of milk thistle in these products.

Eight samples presented red zone(s) due to chlorophyll (Figure 2, orange arrows) and five samples show an unidentified yellow-white zone just above the application position under UV 366 nm prior to derivatization (Figure 2, red arrows).

### 3.2 Coneflower case study

**FIGURE 3:**
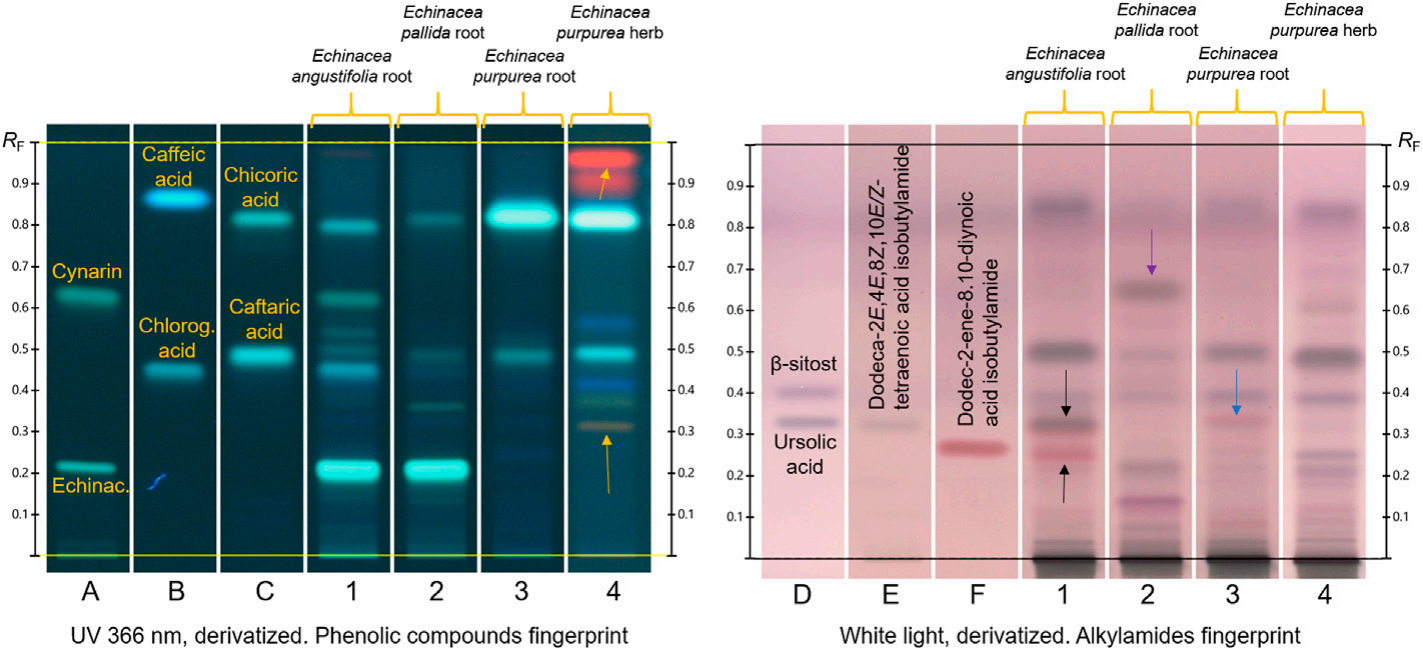
Characteristic HPLTC fingerprints of standards (tracks **A –E**) and coneflower herbal drugs (tracks one–4): phenolic compounds (left image); alkylamides (after second heating step, right image). Yellow arrows: zones characteristic of *Echinacea purpurea* herb; black arrows: zones due to alkylamides of *E. angustifolia* root; purple arrow: zones characteristic of *E. pallida* root; blue arrow: zone due to alkylamides in *E. purpurea* root.

Figure 3 shows the characteristic fingerprints of the three coneflower roots and the purple coneflower herb obtained with the two HPTLC methods. Some HPTLC characteristics allows to clearly distinguish the four herbal drugs. The most prominent are: (1) echinacoside is present in the roots of *E. angustifolia* and *E. pallida* as a very intense zone, but it is absent in the roots and aerial parts of *E. purpurea*; (2) cynarin is present in the roots of *E. angustifolia*, but not in the other three herbal drugs; (3) the zone due to chicoric acid is very intense in the root and aerial parts of *E. purpurea,* far less intense in the root of *E. pallida*, and absent in the root of *E. angustifolia* (although there is a faint zone below that position); and (4) *E. purpurea* aerial parts show red zones due to chlorophylls and some yellow zones due to flavonoids, which are not present in the three roots. Chlorogenic acid appears mainly in the root of *E. angustifolia,* and caftaric acid mainly in the root and aerial parts of *E. purpurea*, but these two compounds are less helpful for discrimination.

The alkylamide fingerprint (Figure 3, right image) of *E. angustifolia* root shows one pink and one brown zone at the positions of dodec-2-ene-8,10-diynoic acid isobutylamide and dodeca-2E,4E,8Z,10*E/Z*-tetraenoic acid isobutylamide, respectively (black arrows). *E. purpurea* root shows the upper alkylamide co-eluting with another pink zone due to an unidentified substance (blue arrow). These zones are absent in the other species and *E. purpurea* aerial parts. *E. pallida* root shows a brown zone absent in all other fingerprints (purple arrow).

**FIGURE 4:**
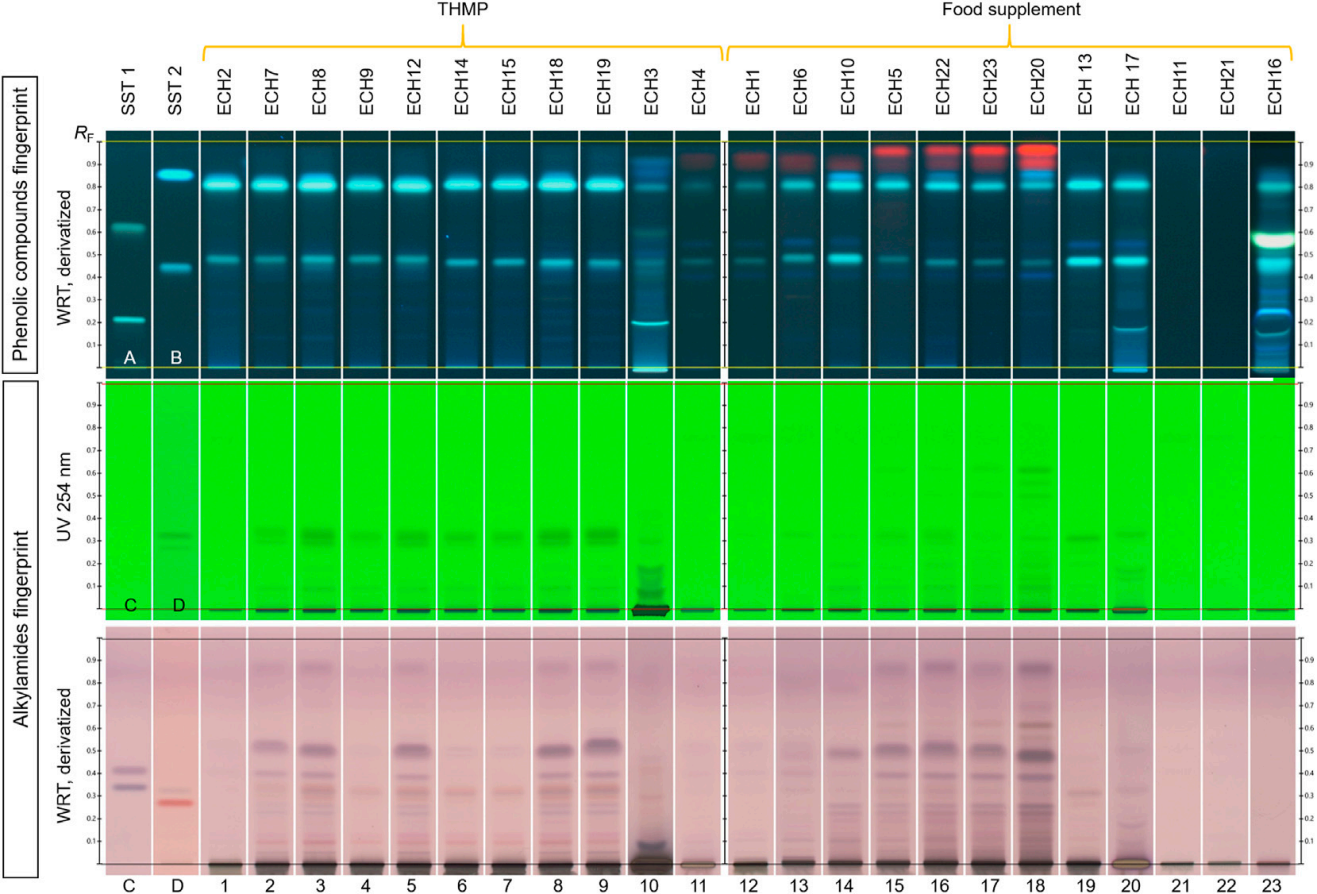
Alkylamide fingerprints of standards and *Echinacea* spp. products, in different detection modes. Tracks **(A)** echinacoside and cynarin, **(B)** chlorogenic and caffeic acids, **(C)** Ursolic acid and β-sitosterol and **(D)** dodeca-2E,4E,8Z,10E/Z-tetraenoic acid isobutylamide and dodec-2-ene-8,10-diynoic acid isobutylamide (with increasing *R*_F_); 1–23: ECH products 1–23.

In Figure 4, products are grouped by regulatory categories. In each group, tracks were arranged based on similarity in visual inspection of the fingerprint of phenolic compounds. All THMP (tracks 1–11) and nine of the twelve food supplement products (tracks 12–18, 20 and 23) show fingerprints compliant with their labels. Regarding their phenolic fingerprint, nine THMP present a composition similar to *E. purpurea* root (tracks 1–9), one THMP shows zones characteristic of *E. angustifolia* root (track 10), one THMP (track 11) and seven food supplement products (tracks 12–18) show zones similar to *E. purpurea* aerial parts. These herbal drugs were declared on the labels. Two food supplement products (tracks 20 and 23), declared to contain *E. purpurea* aerial parts plus *E. angustifolia* root, show no zones characteristic of chlorophyll. Regarding the alkylamides profile, two THMP products (tracks 1 and 11) and four food supplement products (tracks 12, 13, 20 and 23) show very faint zones under UV 254 nm prior to derivatization and under white light after derivatization.

Of the remaining three food supplement samples, two (tracks 21–22) show no zones due to coneflower in either fingerprint. Sample on track 19 declared to contain *E. purpurea* aerial parts but show no zones due to chlorophyll in the phenolic compounds fingerprint. This zone is expected to be seen in tinctures. Furthermore, its alkylamide fingerprint was very faint.

### 3.3 Black cohosh case study

**FIGURE 5:**
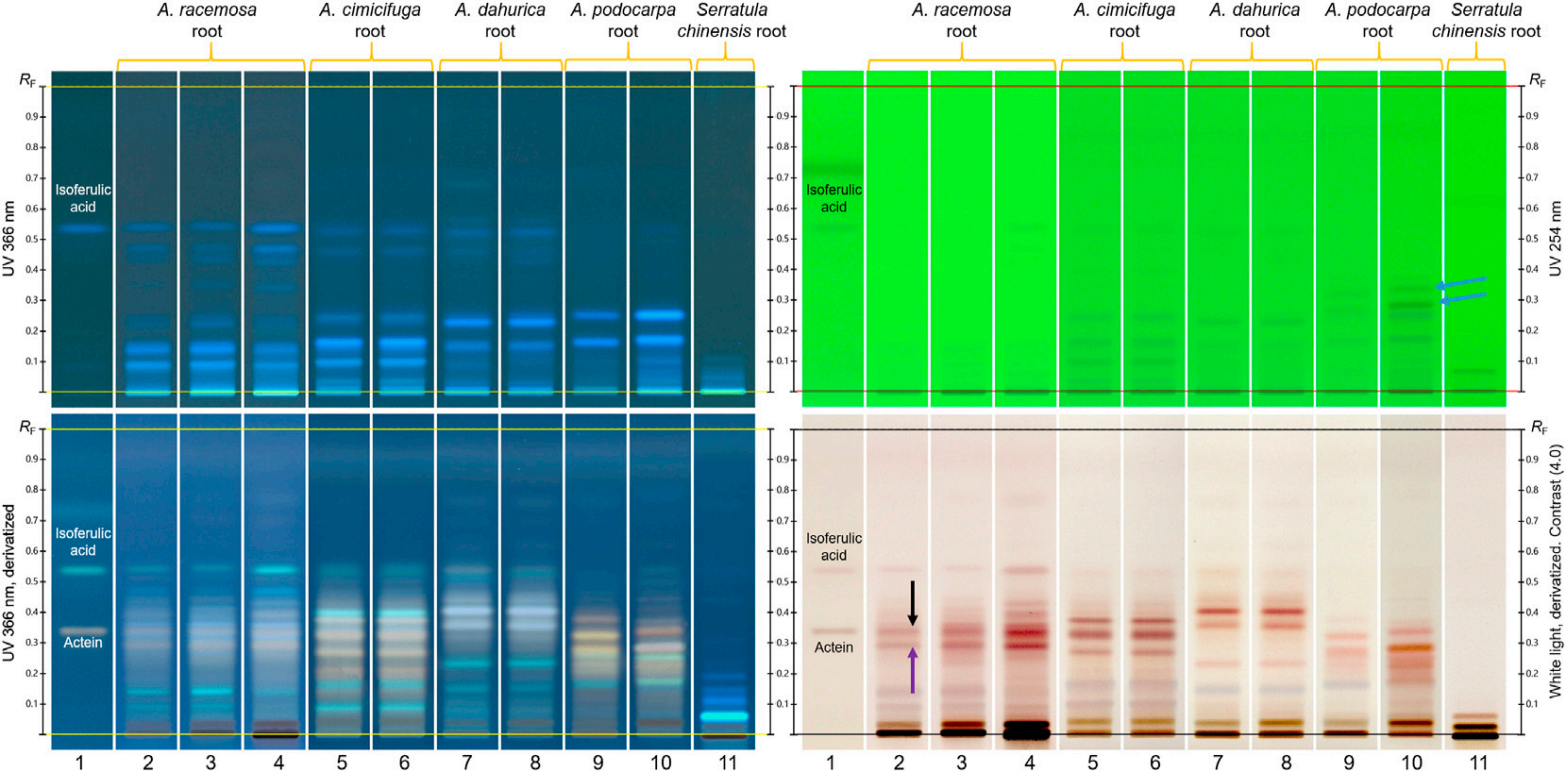
Typical HPTLC fingerprints of *Actaea racemosa* and common adulterants prior to (top) and after derivatization (bottom). Left track in each image shows the chromatogram of the reference substances. Blue arrows: zones characteristic of *A. podocarpa* root; black arrow: zone due to actein in *A. racemosa*; purple arrow: zone due to 26-deoxyactein in *A. racemosa*.

Typical fingerprints of *Actaea cimicifuga*, *A. dahurica*, *A. podocarpa,* and *Serratula chinensis* roots and black cohosh are shown in Figure 5. Under UV 254 nm, *A. podocarpa* (tracks 9–10) shows quenching zones absent in black cohosh and other *Actaea* species (black arrows). Under white light after derivatization, *A. dahurica* (tracks 7–8) lacks zones due to actein (black arrow) and 26-deoxyactein (purple arrow), while *A. cimicifuga* (tracks 5–6) lacks only 26-deoxyactein. Under UV 366 nm after derivatization, all four *Actaea* species show distinctive fingerprints, while *S. chinensis* (track 11) shows mainly an intense blue zone above the application position, absent in the other fingerprints.

**FIGURE 6:**
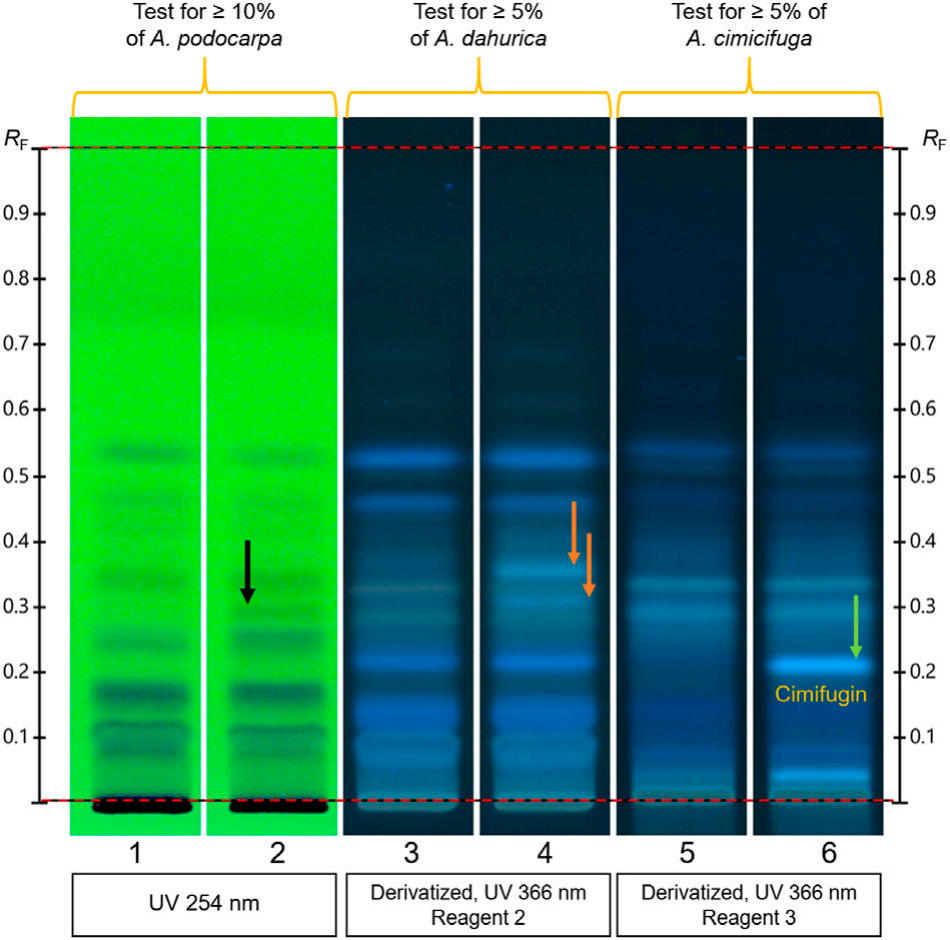
Test for adulteration of *Actaea racemosa* with *A. podocarpa*, *A. dahurica*, and *A. cimicifuga*. Tracks 1, three and 5: *A. racemosa*; tracks 2, four and 6: *A. racemosa* mixed with 10% of *A. podocarpa*, 5% of *A. dahurica* and 5% of *A. cimicifuga*, respectively. Black arrow: zones characteristic of *A. podocarpa* root; orange arrows: zones characteristic of *A. dahurica* root; green arrow: zone due to cimifugin, characteristic of *A. cimicifuga* root.

As shown in Figure 6 (test for other species), adulteration of *A. racemosa* with ≥10% of A*. podocarpa* shows a quenching zone (black arrow, track 2) under UV 254 nm prior to derivatization, absent in pure *A. racemosa* (track 1). Under UV 366 nm after derivatization with antimony trichloride reagent, *A. racemosa* adulterated with ≥5% of A*. dahurica* shows two green zones, one above and one below actein (orange arrows, track 4), absent in pure *A. racemosa* (track 3). Under UV 366 nm after derivatization with boric acid and oxalic acid reagent, *A. racemosa* adulterated with ≥5% of A*. cimicifuga* shows one blue zone due to cimifugin (green arrow, track 6), and another blue zone above the application position. These zones are absent in *A. racemosa* (track 5).

HPTLC fingerprints of black cohosh food supplements and herbal drugs/herbal preparations are presented in supplementary information, Supplementary Figures S1–7. Typical fingerprints are shown in Figure 7.

**FIGURE 7:**
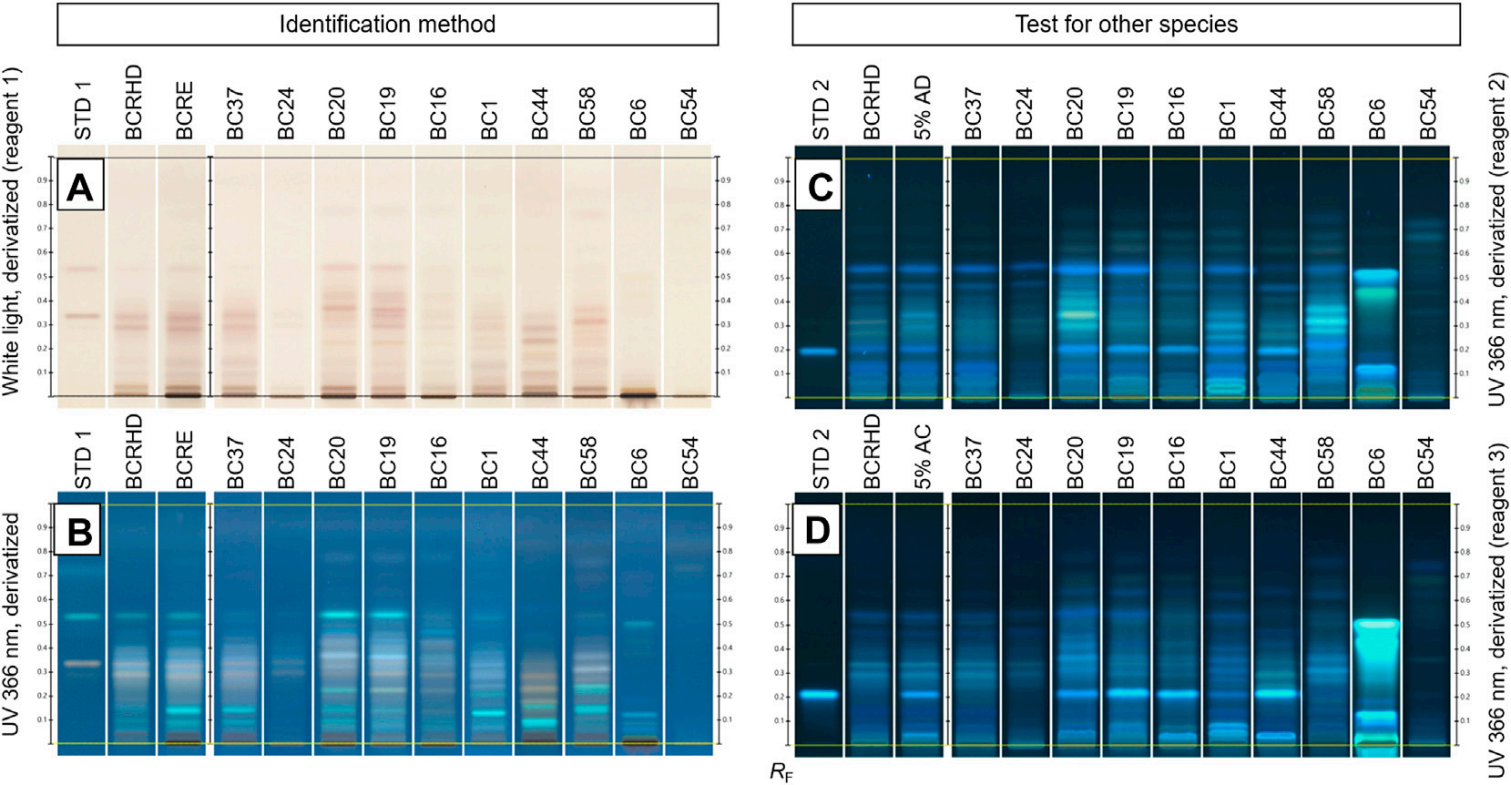
HPTLC fingerprint of 11 black cohosh products and herbal drugs/herbal preparations. STD1: actein and isoferulic acid (increasing R_F_); STD2: cimifugin; RBCR: reference black cohosh root; RBCE: reference black cohosh root extract; 5% AD: *Actaea racemosa* mixed with 5% of *A. dahurica*; 5% AC: *A. racemosa* mixed with 5% of *A. cimicifuga*. Fingerprints **(A,B)** are used for identification of black cohosh root, while fingerprints **(C,D)** are used for detecting adulteration with *A. dahurica* and *A. cimicifuga*, respectively.

Of the sixty food supplement products and herbal drugs/herbal preparations, eighteen food supplements (BC2-5, 7–9, 11, 13–15, 18, 22–24, 26, 30, and 32) and nine herbal drugs/herbal preparations (BC34-40, and 49–50) show fingerprints characteristic of *A. racemosa* (e.g., BC37, Figures 7A, B). Of these samples, fingerprints of eight are fainter than those of reference herbal drug (BCRHD) and reference extract (BCRE) (e.g., BC24, Figures 7A, B). Nevertheless, these samples were considered compliant with their labels concerning identity, but were likely of low potency.

The remaining thirty-three samples are of questionable quality. None of them were concluded to be adulterated with *A. podocarpa* or *S. chinensis*. Fifteen products contained *A. racemosa* adulterated with other *Actaea* species. Of these, seven food supplement products (BC1, 16, 19, 20, 21, 27 and 28) and seven herbal drugs/herbal preparations (BC 45, 48, 51–53, 56–57) show zones due to *A. cimicifuga* and *A. dahurica* in addition to those of *A. racemosa* (e.g., BC16, 19 and 20, Figure 7). Ten of the fifteen samples (BC16, 19, 27, 28, 45, 48, 51–53, 56–57) have the green zones characteristic for adulteration with *A. dahurica* fainter than the 5% accepted by the Ph. Eur. (e.g., BC19 and 16, Figure 7C). Sample BC41 is adulterated with *A. cimicifuga* only.

The other eighteen samples show no traces of black cohosh. Of these, two food supplement products (BC12 and 17) have zones due to *A. dahurica* and *A. cimicifuga* (e.g., similar to BC16, Figures 7C, D). Four food supplement products (BC10, 25, 29, 31) and two herbal drugs/herbal preparations (BC44, 47) present faint fingerprints, in composition similar to that of *A. cimicifuga* (e.g., BC44, Figures 7A, B, D indicating the presence of this species alone. Seven herbal drugs/herbal preparations (BC42, 43, 46, 55, 58–60) contain only *A. dahurica* (e.g., BC58, Figures 7C, D). The three remaining samples (BC6, 33, and 54) show fingerprints different from all *Actaea species* (e.g., BC6, Figures 7A, B) or only barely detectable zones.

## 4 Discussion

Herbal products regulated in different categories can be of very different quality. This fact has been demonstrated by the increasing number of reports of quality issues in various herbal products. These reports are frequently related to accidental and intentional adulterations with chemical substances (including chemical drugs), plant parts (from misidentified plants, fake or inferior plant materials, non-officinal plant parts), extracts, or other materials (e.g. sand) (Zhang et al., 2012; Reynertson and Mahmood, 2015; Parveen et al., 2016; Srirama et al., 2017).

Because quality assessment starts with suitable identification of the herbal ingredient, several problems can already be detected during this step. HPTLC, as recommended for assessing the identity of herbals, can deliver other valuable data for describing the quality. When the new Ph. Eur. chapter two.8.25 (European Directorate for the Quality of Medicines and Healthcare, 2017a) or USP <203> (United States Pharmacopeial Convention, 2017) is followed, the entire fingerprint and additional detection modes could be considered for the evaluation rather than only a few zones, specified by the respective monograph. Detecting the presence or absence of certain zones may indicate quality problems (Cañigueral et al., 2018). This approach is a step towards better exploiting HPTLC data. It was used in three case studies, with the objectives of assessing whether the quality of different herbal drugs, preparations, and products is related to their regulatory category.

Milk thistle fruits (*Silybum marianum* (L.) Gaertn.), coneflower (root and herb of *Echinacea purpurea* (L.) Moench and roots of *E. angustifolia* DC. and *E. pallida* Nutt.) and black cohosh root (*Actaea racemosa* L.) were selected because of their market importance. HPTLC methods for identification were adapted from the Ph. Eur., with the most significant deviation from the original methods being the inclusion of the entire fingerprint (beyond what is prescribed in the Ph. Eur. monographs) into evaluation and interpretation. Moreover, using additional detection modes on the same plate gave access to confirmatory and complementary information without additional analytical efforts. HPTLC chromatograms were evaluated visually. This process depends on the skills and experience of the analyst, but reflects the actual routine use of HPTLC in quality control. To further substantiate the findings, HPTLC fingerprints can be converted into peak profiles and then evaluated quantitatively (Frommenwiler et al., 2018; Frommenwiler et al., 2019). As this goes beyond the requirements of the Pharmacopoeia standards, this step was omitted.

Comparing the analytical results obtained for the individual samples with the table description of the respective Ph. Eur. monograph afforded a classification into good quality samples, which were in compliance with the descriptions and samples of questionable quality, which were not. This procedure was employed regardless of the regulatory category of the samples even though supplements generally do not claim compliance with a monograph. The quality of supplements claiming to contain other than the monographed herbal drug/extract was evaluated taking the complementary information into account.

### 4.1 Milk thistle

*Silybum marianum* fruits have unique macroscopic and chemical characteristics. Therefore, quality problems of milk thistle products are rarely related to substitution or mixing with other species. There is a limited number of publications referring to poor quality and adulteration of milk thistle products. Fenclova et al. (2019) analyzed twenty-six milk thistle food supplements purchased in the United States and the Czech market. Mycotoxins, pesticides or microbiological contamination were detected in all tested preparations. Furthermore, the authors identified significant differences in the silymarin content between the products, often contradicting the information provided on the labels.

**FIGURE 8:**
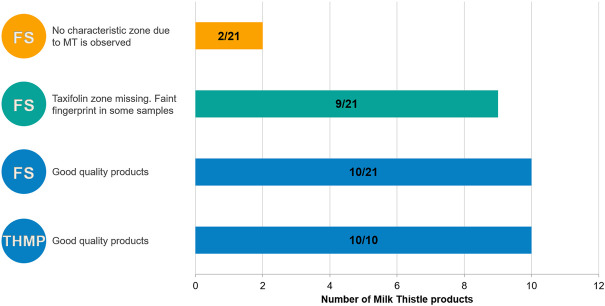
Summary of the quality of milk thistle products: number of samples/total products per category. THMP: Traditional Herbal Medicinal Product; FS: Food supplement.

Products analyzed in the present study claimed to contain different plant parts of *S. marianum*, the herbal drug and/or its extracts. HPTLC of flavonolignans and flavonoids can differentiate between these ingredient types. Evaluation of quality is summarized in Figure 8. Only food supplement samples (11 out of 21) had questionable quality. Nine of them show no zone at the position of taxifolin, but only the presence of the silymarin zones. Those samples were not considered to be of good quality because they lack one of the zones described in the HPTLC identity test of the Ph. Eur. monograph. The other two samples show no zone characteristic of milk thistle, confirming the absence of that herbal drug in the product.

Fingerprints of some samples of both, good and questionable quality, feature additional zones that are not characteristic of milk thistle, but related to other declared ingredients in the product. The presence of those zones could be caused by declared milk thistle herb, spirulina (biomass of *Arthrospira platensis* and *A. maxima*), alfalfa (*Medicago sativa* L.), artichoke (*Cynara cardunculus* L.), dandelion (*Taraxacum officinale* F.H.Wigg.), boldo (*Peumus boldus* Molina) and peppermint leaf (*Mentha* × *piperita* L.). The unidentified yellow-white zone just above the application position in five samples could indicate a quality issue. Common excipients were ruled out as source of this zone by additional experiments. The origin of this zone remains unclear.

### 4.2 Coneflower

The employed set of HPTLC methods for identification of coneflower, published by the HPTLC Association (HPTLC Association, 2014), were based on the existing Ph. Eur. monographs for coneflower herbal drugs. They include detection of phenolic compounds and alkylamides. Some parameters were optimized, such as application volume, developing distance, and detection modes. Both tests provide specific outcomes, useful for distinguishing plant parts and species of coneflowers. In addition, the absense of specific zones in the alkylamides fingerprint can indicate whether poor quality herbal starting materials were used. According to Perry et al. (2000), the levels of alkylamides in *E. purpurea* root can be significantly reduced when it is stored at room temperature for up to 64 weeks. Therefore, the fingerprint of an old, incorrectly stored herbal ingredient, may not contain alkylamide zones.

**FIGURE 9:**
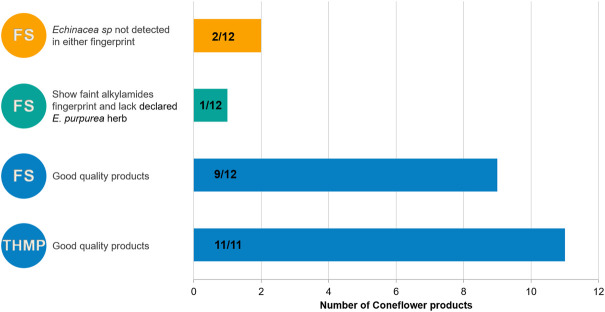
Summary of the quality of coneflower products: number of samples/total products per category. THMP: Traditional Herbal Medicinal Product; FS: Food supplement.

In our analyses, only food supplement samples (3 out of twelve) exhibited questionable quality due to lack of zones characteristic of *E. purpurea* herb, faint alkylamides fingerprint or lack of zones characteristic of *E. spp*. All THMP and nine food supplement products were of good quality. A summary of the quality of coneflower products is presented in Figure 9.

Two food supplement products (ECH 17 and 16) declared to contain water-glycerin extracts of *E. purpurea* aerial parts but showed no zones characteristic of chlorophyll. The use of glycerin and water could have prevented the extraction of chlorophyll. Therefore, they were also considered compliant with their labels.

Alkylamide profiles of two THMP products (ECH 2 and 14) and four food supplement products (ECH 1, 6, 11 and 16) show very faint zones. The possible reasons may be that: (1) the extract used in samples ECH 2, 11 and 16 were prepared using low percentages of ethanol (e.g., 30%) or water/glycerin, leading to lesser amounts of lower-polarity constituents, such as alkylamides; or (2) due to lack of information regarding the drug/extract ratio for samples ECH 1, 6 and 14, the selected sample preparation may not have been optimal thus yielding overall faint fingerprints.

### 4.3 Black cohosh

The HPTLC identification method for black cohosh of the Ph. Eur. (European Directorate for the Quality of Medicines and Healthcare, 2017b) is capable of discriminating the four main adulterants: *A. cimicifuga*, *A. dahurica*, *A. podocarpa,* and *Serratula chinensis* root. However, that method is not suitable for detecting the presence of the adulterants in a mixture.

Therefore, Ankli et al. (2008) proposed another HPTLC test for detection of adulteration in mixtures, which was incorporated into the monograph as part of the tests section under the title “Adulteration with *Cimicifuga americana* Michx., *C. foetida* L., *C. dahurica* (Turcz.) Maxim. or *C. heracleifolia* Kom”. In this test, 20 µL of samples are applied twice onto the plate (once on each half. After development, the plate is cut in the middle. One half is derivatized with antimony trichloride reagent (reagent 2), and the other half with boric acid and oxalic acid reagent (reagent 3). Those derivatization reagents highlight special features of the adulterants, not seen with sulfuric acid.

**FIGURE 10:**
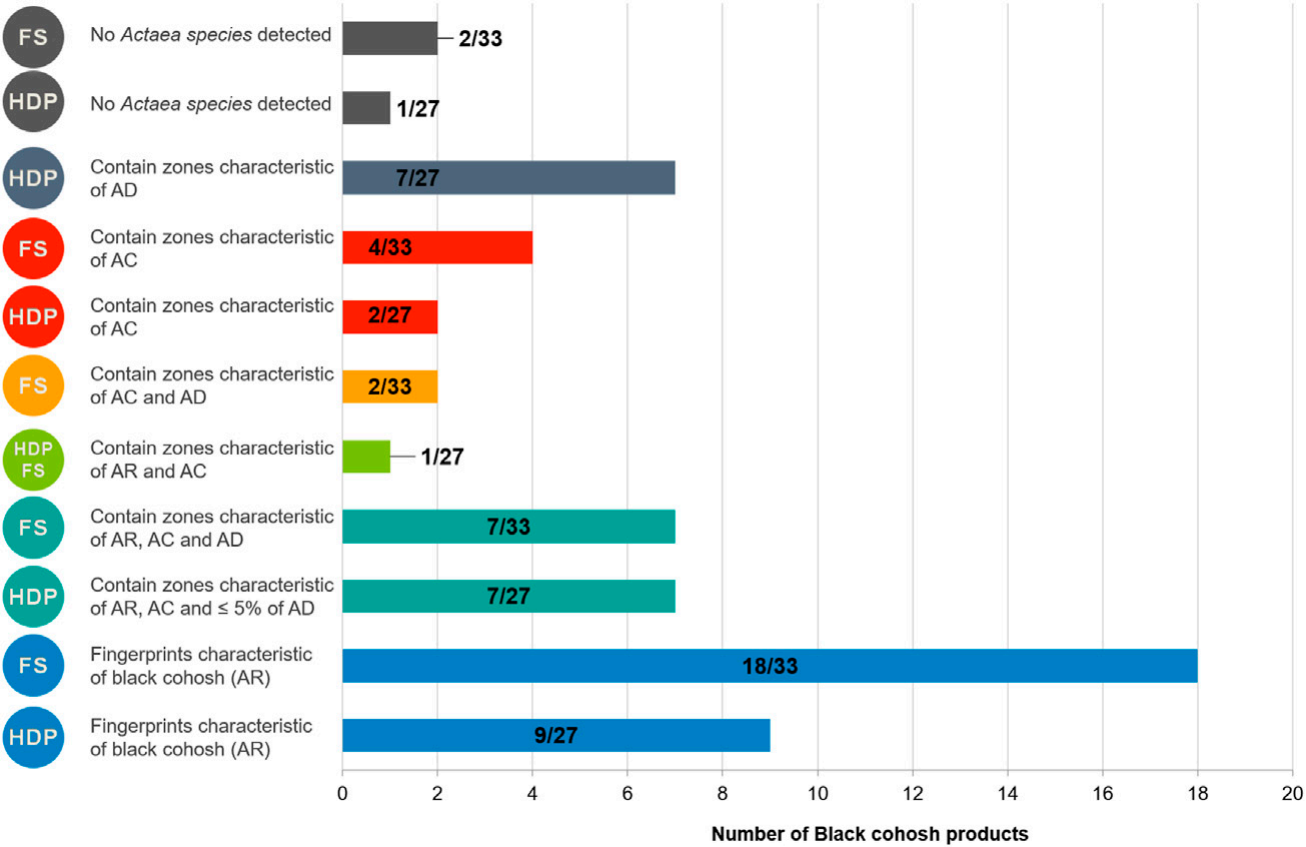
Summary of the quality of black cohosh food supplement (FS) and herbal drugs and preparations (HDP): number of samples showing the concerned characteristic/total products per category. AC: *Actaea cimicifuga*; AD: *A. dahurica*; AR: *A. racemosa*.

After submitting black cohosh samples to those three analyses, more than half were found to be adulterated. A summary of the quality of those samples is presented in Figure 10. The main problems encountered were: presence of one or more adulterants (15 samples), replacement by one or more adulterants (15 samples), fingerprints different from all *Actaea species* or only very faint zones detected (3 samples).

## 5 Conclusion

The three case studies demonstrate that evaluating the entire HPTLC fingerprints in several detection modes is very helpful for uncovering adulterations and other quality issues in herbal drugs, preparations and products.

Significant quality differences were observed between the tested products commercialised under different regulatory frameworks (Table 5). All products marketed as medicinal products were compliant with label statements for the parameters investigated here. Their fingerprints were consistent, without unexpected zones observed. This was not the case with food/dietary supplement products. Quality issues were found in 52% of milk thistle products, 25% of coneflowers products, and 46% of black cohosh food supplement products. Also, 67% of herbal drugs and herbal preparations labelled as black cohosh presented quality issues. Quality issues found included absence of the herbal ingredient declared on the product label, lower concentration of the herbal ingredient per unit of pharmaceutical form, and presence of adulterants, especially related species. The results obtained in the present work support the theory that less stringent regulations can negatively affect the quality of herbal products.

**TABLE 5:** Classification of the samples as having good or questionable quality, grouped according to their regulatory status. Percentages are given in relation to the total number of samples of each category and the absolute number of samples in parenthesis.

	Milk thistle fruit	Coneflower	Black cohosh root
Traditional Herbal Medicinal Products			
Good quality samples	100% (10)	100% (11)	─
Questionable quality	0% (0)	0% (0)	─
Food supplements			
Good quality samples	47.6% (10)	75% (9)	54.5% (18)
Questionable quality	52.4% (11)	25% (3)	45.5% (15)
Herbal drugs/herbal preparations			
Good quality samples	─	─	33.3% (9)
Questionable quality	─	─	66.6% (18)

The cost for the HPTLC analysis per sample, if only one sample were to be analyzed on one plate and in one run, is approximately $46 for coneflower (because two methods were used), and $21 for black cohosh and milk thistle. A detailed calculation of the cost is shown in the supplementary information. It is important to highlight that the analysis cost per sample is reduced drastically when more than one sample is analyzed on the same plate.

HPTLC using optimized and standardized methods and evaluating the entire fingerprint in several detections, proves to be a cost-efficient technique for proper identification and quick detection of a range of quality issues in herbal drugs, preparations and products.

## Data Availability

The original contributions presented in the study are included in the article/Supplementary Material further inquiries can be directed to the corresponding author.
