# Supplementary material for: Investigation of market herbal products regulated under different categories: How can HPTLC help to detect quality problems?
Source: Front Pharmacol. 2022 Aug 8;13:925298. doi: 10.3389/fphar.2022.925298 (PMC9393483; doi:10.3389/fphar.2022.925298)
Supplement: Supplementary file 1 [file Table1.DOCX]

Supplementary information

Investigation of market herbal products regulated under different categories: how can HPTLC help to detect quality problems?

Débora A. Frommenwilera^1,2*^, Eike Reich^1^, Maged H.M. Sharaf ^3^, Salvador Cañigueral^2^ and Christopher J. Etheridge^4^

^1^CAMAG Laboratory, Muttenz, Switzerland;

^2^Unit of Pharmacology, Pharmacognosy and Therapeutics, Faculty of Pharmacy and Food Sciences, University of Barcelona, Barcelona, Spain

^3^HPTLC Association, Dianastrasse 10, CH4310, Rheinfelden Switzerland

^4^British Herbal Medicine Association (BHMA), Exeter, UK

*** Correspondence:**Débora A. Frommenwiler
debora.frommenwiler@camag.com

**Table S1** Description of the Milk thistle products analysed in this work, their dosage form and regulatory status. MTE: Milk thistle extract; MTS: Milk thistle seed; MTHP: Milk thistle herb powder; MTT: Milk thistle tincture; THMP: traditional herbal medicinal product.

| **Sample N°** | **Description of products per dosage unit** | **Dosage form** | **Regulatory status** |
| --- | --- | --- | --- |
| MT1 | MTE 300 mg equivalent to 7.2-8.1 g of fruit (DER: 24-27:1). Standardized to contain 174 mg of silymarin. Extraction solvent: Acetone 95% | Tablet | THMP |
| MT2 | MTE 137.5-165 mg equivalent to 2.75-6.60 g of fruit (DER: 20-40:1). Standardized to contain 82.5 mg of silymarin. Extraction solvent: Ethyl acetate | Tablet | THMP |
| MT3 | MTE 100 mg equivalent to 3 g of fruit (DER: 30:1). Standardized to contain 80 mg of silymarin. | Tablets | Food supplement |
| MT4 | MTS 450 mg | Capsules | THMP |
| MT5 | MTE 83.3 mg equivalent to 3 g of herbal drug (DER: 36:1). Standardized to contain 66.67 mg of silymarin. | Tablets | Food supplement |
| MT6 | MTE 100 mg equivalent to 4 g of herbal drug (DER: 40:1), MTHP 325 mg and MTS 25 mg. Each dosage form is standardized to contain 80 mg of silymarin | Tablets | Food supplement |
| MT7 | MTE 83.3 mg equivalent to 3 g of herbal drug (DER: 36:1). Standardized to contain 66.67 mg of silymarin. | Tablets | Food supplement |
| MT8 | MTE 100 mg standardized to contain 80 mg of silymarin, and MT aerial parts and seeds powder 350 mg | Capsule | Food supplement |
| MT9 | MTE 300 mg equivalent to 7.2-8.1 g of fruit (DER: 24-27:1). Standardized to contain 174 mg of silymarin. Extraction solvent: Acetone 95% | Tablets | THMP |
| MT10 | MTE equivalent to 485 mg of milk thistle complex tincture. Other ingredients: artichoke leaves 46%, dandelion herb 12%, boldo leaf 7% and peppermint leaf 3%. | Tablets | Food supplement |
| MT11 | MTE 86 mg equivalent to 3 g of seeds (DER: 35:1). Standardized to contain 68 mg of silymarin. | Tablets | Food supplement |
| MT12 | MTE 40 mg standardized to 32 mg of silymarin. Other ingredients: dandelion root 100 mg, curcumin extract 25 mg, artichoke leaf powder 50 mg | Tablets | Food supplement |
| MT13 | MTE 40 mg standardized to 32 mg of silymarin. Other ingredients: dandelion root 100 mg, curcumin extract 25 mg, artichoke leaf powder 50 mg | Tablets | Food supplement |
| MT14 | MT dried fruit complex tincture 32%, artichoke leaves 46%, dandelion herb 12%, boldo leaf 7% and peppermint leaf 3%. Extraction solvent: alcohol 62% | Tincture | Food supplement |
| MT15 | MTE 125 mg equivalent to 2.5-5 g of fruit (DER: 20-40:1). Standardized to contain 62.5-75 mg of silymarin. | Tablets | THMP |
| MT16 | MT seeds 500 mg. Standardized to contain 15-25 mg of silymarin | Capsules | Food supplement |
| MT17 | MT seeds 400 mg | Capsules | Food supplement |
| MT18 | MTT 97.5 mg of tincture per mL, equivalent to 1 g of MT seed | Tincture | Food supplement |
| MT19 | MTT 1 mL of tincture is equivalent to 856 mg of MT fresh seed | Tincture | Food supplement |
| MT20 | MT dried fruit complex tincture 32%, artichoke leaves 46%, dandelion herb 12%, boldo leaf 7% and peppermint leaf 3%. Extraction solvent: alcohol 62% | Tincture | Food supplement |
| MT21 | MTE 100 mg equivalent to 3 g of fruit (DER: 30:1) | Tablets | Food supplement |
| MT22 | MTE 175 mg, MTHP 172 mg. Other ingredients: spirulina and alfalfa | Capsules | Food supplement |
| MT23 | 1.5 g of a blend of herbal ingredients and MT | Tincture | Food supplement |
| MT24 | MTE of seeds 100 mg standardized to contain 80 mg of silymarin and 350 mg of powdered MT seeds and aerial parts | Capsules | Food supplement |
| MT25 | MTE 193-261 mg equivalent to 3.725-10.818 g of fruit (DER: 19.3-41.4:1). Standardized to contain 108 mg of silymarin. Extraction solvent: Acetone 95% | Capsules | THMP |
| MT26 | MTE 89-121 mg equivalent to 1.721-5.0 g of fruit (DER: 19.3-41.3:1). Standardized to contain 50 mg of silymarin. Extraction solvent: Acetone 95% | Capsules | THMP |
| MT27 | MTE 193-261 mg equivalent to 3.725-10.818 g of fruit (DER: 19.3-41.4:1). Standardized to contain 108 mg of silymarin. Extraction solvent: Acetone 95% | Capsules | THMP |
| MT28 | MTE 89-121 mg equivalent to 1.721-5.0 g of fruit (DER: 19.3-41.3:1). Standardized to contain 50 mg of silymarin. Extraction solvent: Acetone 95% | Capsules | THMP |
| MT 29 | MTE 100 mg equivalent to 4 g of fruit (DER: 40:1). Standardized to contain 80 mg of silymarin. MT seeds and aerial parts 350 mg | Capsule | Food supplement |
| MT 30 | MTE seed 175 mg standardized to contain 140 mg of silymarin. Blessed thistle stem, leaf and flower, 120 mg | Capsule | Food supplement |
| MT 31 | MTE 133-324 mg equivalent to 3.325-9.720 g of fruit (DER: 25-30:1). Standardized to contain 108 mg of silymarin. Extraction solvent: Acetone 99% | Capsule | THMP |

**Table S2** Description of the Echinacea products analyzed in this work, their dosage form and regulatory status. ECHE: Echinacea dried extract; ECHT: Echinacea tincture; ECHHD: Echinacea herbal drug; THMP: traditional herbal medicinal product.

| **Sample N°** | **Description of products per dosage unit** | **Dosage form** | **Regulatory status** |
| --- | --- | --- | --- |
| ECH1 | ECHE 380 mg from fresh *E. purpurea* herb (DER: 7.5-14.6:1), ECHE 20 mg from fresh *E. purpurea* root (DER: 7.1-12.5:1) Extraction solvent: ethanol 65% | Tablets | Food supplement |
| ECH2 | ECHE 70 mg from fresh *E. purpurea* root equivalent to 460-530 mg of root (DER: 6.6-7.6:1) Extraction solvent: ethanol 30% | Tablets | THMP |
| ECH3 | ECHE 128 mg of *E. angustifolia* (part of the herbal drug not declared) per mL of extract (DER: 1:1). Extraction solvent: ethanol 45%. Other ingredients: wild indigo (35 mg of extract DER 1:1, per mL) and fulmitory (32 mg of extract DER 1:1, per mL) | Oral Liquid | THMP |
| ECH4 | ECHE 380 mg from fresh *E. purpurea* herb (DER: 12:1), ECHE 20 mg from fresh *E. purpurea* root (DER: 11:1). Extraction solvent: ethanol 65% | Chewable Tablets | THMP |
| ECH5 | ECHE 65 mg from *E. purpurea* root (DER: 4:1) and ECHHB 265 mg of *E. purpurea* aerial parts | Capsules | Food supplement |
| ECH6 | ECHT 860 mg of *E. purpurea* herb per mL of tincture (DER: 7.5-14.6:1). ECHT 45 mg of *E. purpurea* root per mL of tincture (DER: 7.1-12.5:1) Extraction solvent: ethanol 65% | Oral Liquid | Food supplement |
| ECH7 | ECHE 70 mg from fresh *E. purpurea* root equivalent to 420-560 mg of root (DER: 6-8:1) Extraction solvent: ethanol 75% | Tablets | THMP |
| ECH8 | ECHE 140 mg from fresh *E. purpurea* root equivalent to 838-1177 mg of root (DER: 6-8.4:1) Extraction solvent: ethanol 75% | Capsules | THMP |
| ECH9 | ECHE 71.5 mg from fresh *E. purpurea* root equivalent to 429-500 mg of root (DER: 6-7:1) | Tablets | THMP |
| ECH10 | ECHT 250 mg of *E. purpurea* herb per mL of tincture (DER: 5:1). Extraction solvent: ethanol 66% | Oral Liquid | Food supplement |
| ECH11 | ECH standardized extract 160 mg equivalent to 3200 mg of herb (DER: 45:1). Note: 160 mg of extract is equivalent to 7200 mg of fresh herb and not 3200 mg, as declared in the label. | Tablets | Food supplement |
| ECH12 | ECHE 56 mg from fresh *E. purpurea* root equivalent to 338-450 mg of root (DER: 6-7:1) Extraction solvent: ethanol 75% | Tablets | THMP |
| ECH13 | ECHT 480 mg of *E. purpurea* herb per mL of tincture | Oral Liquid | Food supplement |
| ECH14 | ECHE 71.5 mg from fresh *E. purpurea* root equivalent to 429-500 mg of root (DER: 6-7:1) | Tablets | THMP |
| ECH15 | ECHE 71.5 mg from fresh *E. purpurea* root equivalent to 429-500 mg of root (DER: 6-7:1) | Tablets | THMP |
| ECH16 | ECHT 1 mL contains 1 g of *E. purpurea* aerial parts and root extract (standardized to 10 mg of phenolic compounds), *E. angustifolia* root extract and Goldenseal root extract. Prepared in Glycerin and water | Tincture | Food supplement |
| ECH17 | ECHT 2 mL contain 325 mg of *E. purpurea* flowers and herbs extract and *E. angustifolia* roots extract equivalent to 1470 mg of herbal drug. Prepared in Glycerin and water | Tincture | Food supplement |
| ECH18 | ECHE 140 mg from fresh *E. purpurea* root equivalent to 838-1177 mg of root (DER: 6-8.4:1) Extraction solvent: ethanol 75% | Capsules | THMP |
| ECH19 | ECHE 105 mg from fresh *E. purpurea* root equivalent to 630-840 mg of root (DER: 6-8:1) Extraction solvent: ethanol 75% | Tablets | THMP |
| ECH20 | ECHHD 400 mg of *E. purpurea* aerial parts | Capsules | Food supplement |
| ECH21 | ECHE equivalent to 1000 mg of *E. purpurea* herb | Tablets | Food supplement |
| ECH22 | ECHE 65 mg from *E. purpurea* root (DER: 4:1) and ECHHB 265 mg of *E. purpurea* aerial parts | Capsules | Food supplement |
| ECH23 | ECHE 65 mg from *E. purpurea* root (DER: 4:1) and ECHHB 265 mg of *E. purpurea* aerial parts | Capsules | Food supplement |

**Table S3** Description of the Black cohosh products analyzed in this work, their dosage form and products type BC: Black cohosh (A. racemosa); BCE: Black cohosh root extract; DS: dietary(food) supplement; HDP: herbal drug or herbal preparation; DER: Drug extract ratio.

| **Sample N°** | **Description of products per dosage unit** | **Dosage form** | **Product type** |
| --- | --- | --- | --- |
| BC1 | BC 1.25 g of root cut / dosage form | Tea bag | Finished product, DS |
| BC2 | BC 540 mg of root powder / dosage form | Capsules | Finished product, DS |
| BC3 | BC 540 mg of root powder / dosage form | Capsules | Finished product, DS |
| BC4 | BC 540 mg of root powder / dosage form | Capsules | Finished product, DS |
| BC5 | BC 540 mg of root powder / dosage form | Capsules | Finished product, DS |
| BC6 | BC 410 mg of root powder / dosage form | Capsules | Finished product, DS |
| BC7 | BC 300 mg of root powder / dosage form. Another ingredient: brown rice powder | Capsules | Finished product, DS |
| BC8 | BC 540 mg of root powder / dosage form | Capsules | Finished product, DS |
| BC9 | BC 540 mg of root powder / dosage form | Capsules | Finished product, DS |
| BC10 | BC 540 mg of root powder / dosage form | Capsules | Finished product, DS |
| BC11 | BC 100 mg of root powder / dosage form | Capsules | Finished product, DS |
| BC12 | BC 540 mg of root powder / dosage form | Capsules | Finished product, DS |
| BC13 | BC 370 mg of root powder / dosage form | Capsules | Finished product, DS |
| BC14 | BC 540 mg of root powder / dosage form | Capsules | Finished product, DS |
| BC15 | BC 200 mg of root powder / dosage form | Capsules | Finished product, DS |
| BC16 | BC 540 mg of root powder / dosage form | Capsules | Finished product, DS |
| BC17 | BC 530 mg of root powder and BCE 20 mg. Extract standardized to 2.5% of triterpene glycosides. Another ingredient: rice flour | Capsules | Finished product, DS |
| BC18 | BC root powder and BCE (545 mg) | Capsules | Finished product, DS |
| BC19 | BC 185 mg of root powder and BCE 40 mg. Extract standardized to 2.5% of triterpene glycosides | Capsules | Finished product, DS |
| BC20 | BC 380 mg of root powder and BCE 80 mg. Extract standardized to 2.5% of triterpene glycosides. Other ingredients: triglycerides, vitamin E and rosemary oil | Capsules | Finished product, DS |
| BC21 | BC 380 mg of root powder and BCE 80 mg. Extract standardized to 2.5% of triterpene glycosides. Other ingredients: triglycerides, vitamin E and rosemary oil | Capsules | Finished product, DS |
| BC22 | BC root powder and BCE (545 mg) | Capsules | Finished product, DS |
| BC23 | BCE 100 mg, standardized to 2.5% of triterpene glycosides. Other ingredients: rice flour | Capsules | Finished product, DS |
| BC24 | BCE 20 mg, standardized to 2.5% of triterpene glycosides, calculated as 26-deoxyactein | Tablet | Finished product, DS |
| BC25 | BCE 40 mg, standardized to 2.5% of triterpene glycosides. Other ingredients: rice flour | Capsules | Finished product, DS |
| BC26 | BCE 40 mg, standardized to 2.5% of triterpene glycosides, calculated as 26-deoxyactein | Capsules | Finished product, DS |
| BC27 | BCE 40 mg, standardized to 8% of triterpene glycosides, calculated as 26-deoxyactein | Capsules | Finished product, DS |
| BC28 | BCE 135 mg (DER: 4:1) and BCE 40 mg, standardized to 2.5% of triterpene glycosides | Capsules | Finished product, DS |
| BC29 | BCE 80 mg, standardized to 2.5% of triterpene glycosides. Other ingredients: rice flour | Capsules | Finished product, DS |
| BC30 | BCE 80 mg, standardized to 2.5% of triterpene glycosides, calculated as 26-deoxyactein | Tablet | Finished product, DS |
| BC31 | BCE 40 mg, standardized to 2.5% of triterpene glycosides, calculated as 26-deoxyactein | Capsules | Finished product, DS |
| BC32 | BCE 250 mg, standardized to 2.5% of triterpene glycosides | Capsules | Finished product, DS |
| BC33 | BCE 80 mg, standardized to 2.5% of triterpene glycosides, calculated as 26-deoxyactein. Other ingredients: natural color | Fluid extract | Finished product, DS |
| BC34 | BC root cut (plant material) | ---- | HDP |
| BC35 | BC root cut (plant material) | ---- | HDP |
| BC36 | BC root cut (plant material) | ---- | HDP |
| BC37 | BC root cut (plant material) | ---- | HDP |
| BC38 | BC root cut (plant material) | ---- | HDP |
| BC39 | BC root cut (plant material) | ---- | HDP |
| BC40 | BC root powder (plant material) | ---- | HDP |
| BC41 | BC root whole (plant material) | ---- | HDP |
| BC42 | BC root whole (plant material) | ---- | HDP |
| BC43 | BC root whole (plant material) | ---- | HDP |
| BC44 | BC root whole (plant material) | ---- | HDP |
| BC45 | BCE powder extract, standardized to 2.5% of triterpene glycosides | ---- | HDP |
| BC46 | BCE powder (not labeled but macroscopically similar to extract) | ---- | HDP |
| BC47 | BCE powder (DER 4:1) | ---- | HDP |
| BC48 | BC root powder (plant material) | ---- | HDP |
| BC49 | BC root powder (plant material) | ---- | HDP |
| BC50 | BC root powder (plant material) | ---- | HDP |
| BC51 | BCE, standardized to 2.5% of triterpene glycosides | ---- | HDP |
| BC52 | BCE, standardized to 2.5% of triterpene glycosides | ---- | HDP |
| BC53 | BC root powder (plant material) | ---- | HDP |
| BC54 | BC root powder (plant material) | ---- | HDP |
| BC55 | BC root whole (plant material) | ---- | HDP |
| BC56 | BCE powder (DER 10:1) | ---- | HDP |
| BC57 | BCE powder (5%) | ---- | HDP |
| BC58 | BC root whole (plant material) | ---- | HDP |
| BC59 | BC root whole (plant material) | ---- | HDP |
| BC60 | BC root whole (plant material) | ---- | HDP |


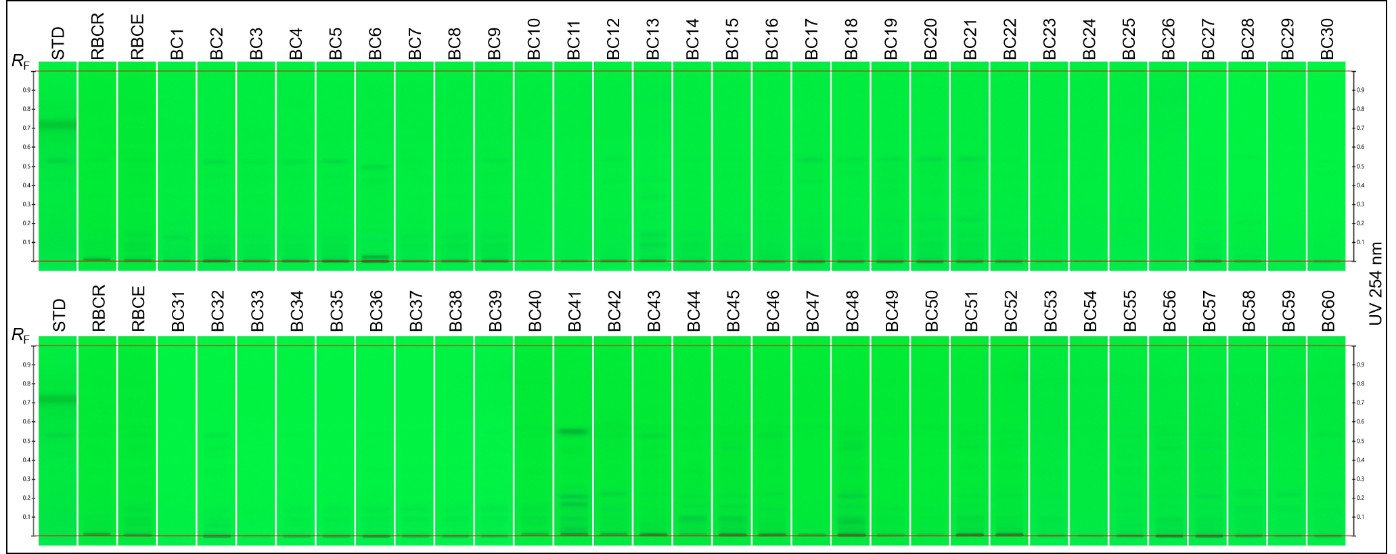


**Figure S1** HPTLC fingerprint of all black cohosh products (BCP) and ingredients (BCI) under UV 254 nm prior to derivatization. Identification method. STD: isoferulic acid; RBCR: reference black cohosh root; RBCE: reference black cohosh root extract.


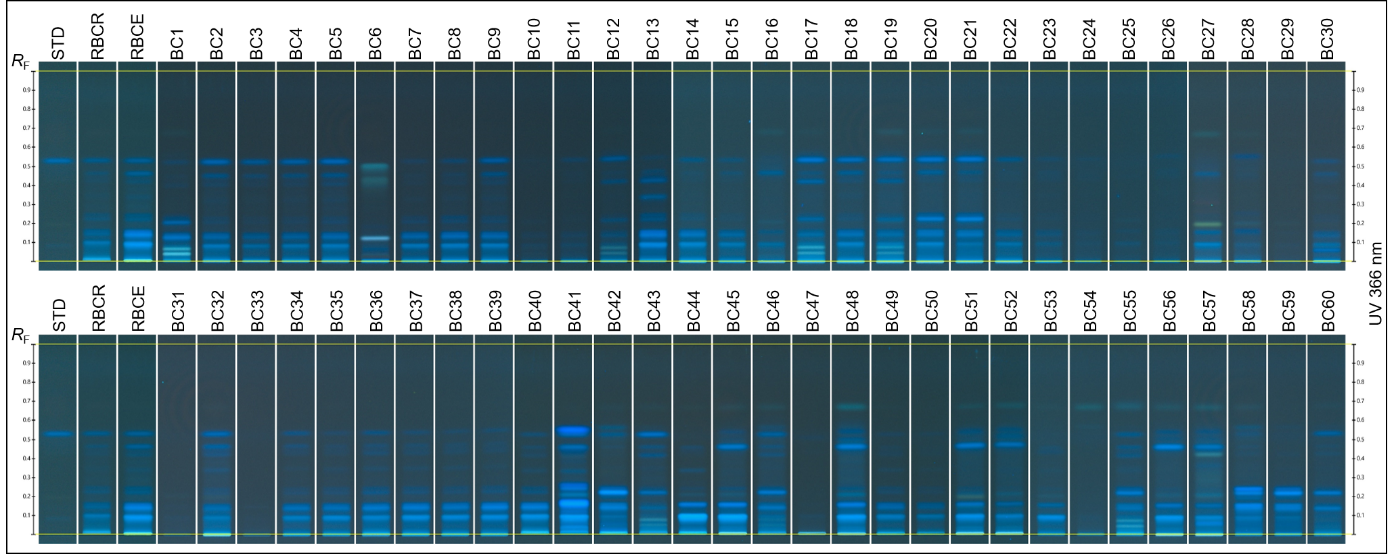


**Figure S2** HPTLC fingerprint of all black cohosh products (BCP) and ingredients (BCI) under UV 366 nm prior to derivatization. Identification method. STD: isoferulic acid; RBCR: reference black cohosh root; RBCE: reference black cohosh root extract.


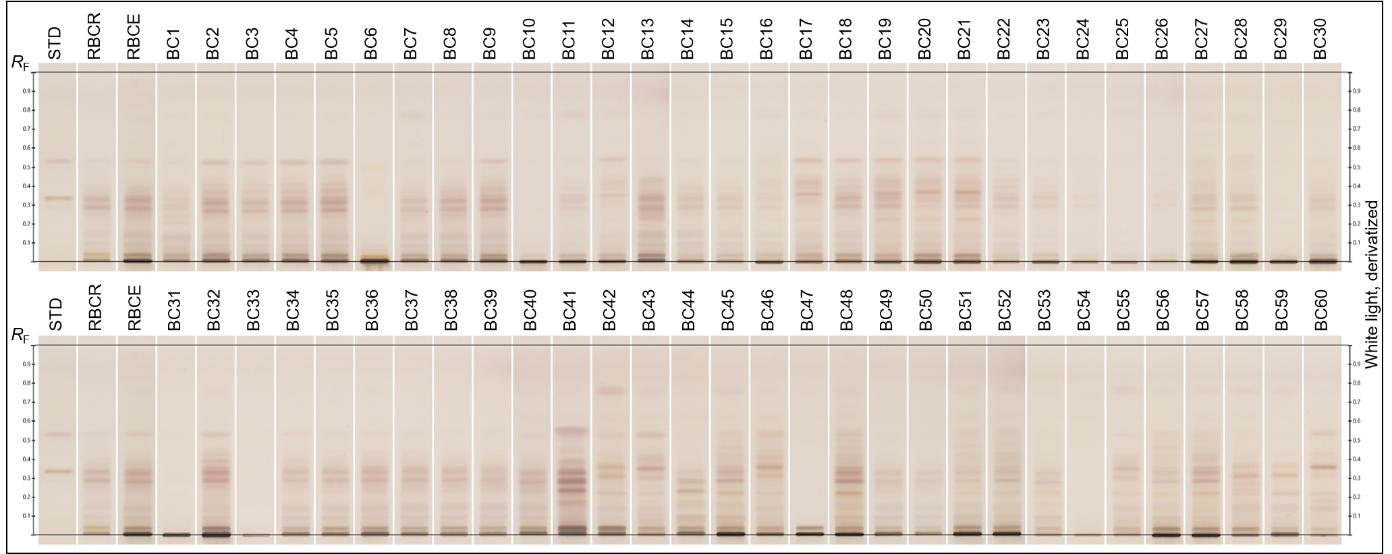


**Figure S3** Identification method. STD: actein and isoferulic acid (increasing R_F_); RBCR: reference black cohosh root; RBCE: reference black cohosh root extract.


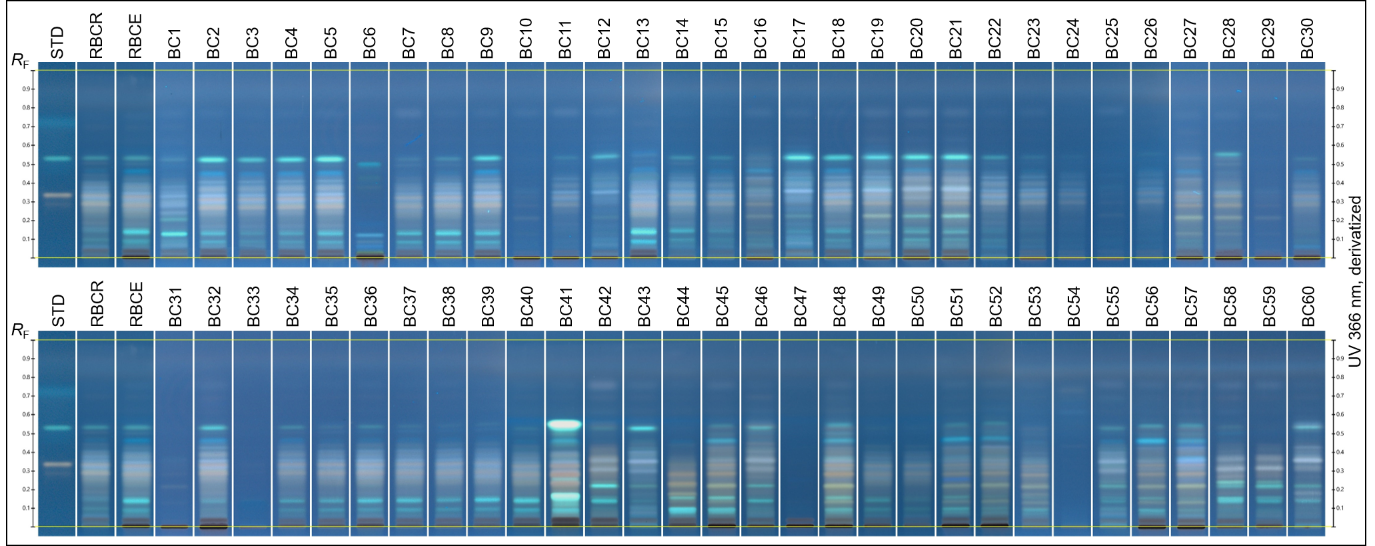


**Figure S4** HPTLC fingerprint of all black cohosh products (BCP) and ingredients (BCI) under UV 366 nm after derivatization with sulfuric acid. Identification method. STD: actein and isoferulic acid (increasing R_F_); RBCR: reference black cohosh root; RBCE: reference black cohosh root extract.


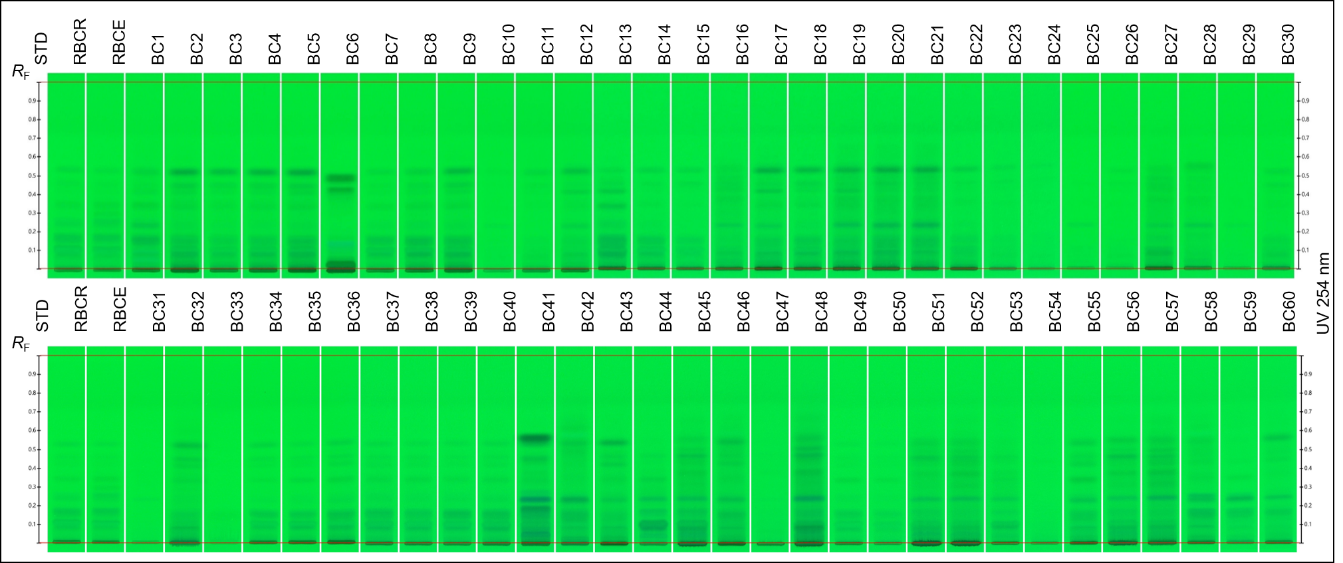


**Figure S5** HPTLC fingerprint of all black cohosh products (BCP) and ingredients (BCI) under UV 254 nm prior to derivatization. Test for A. podocarpa; RBCR: reference black cohosh root; 10% AP: A. racemosa mixed with 10% of A. podocarpa.


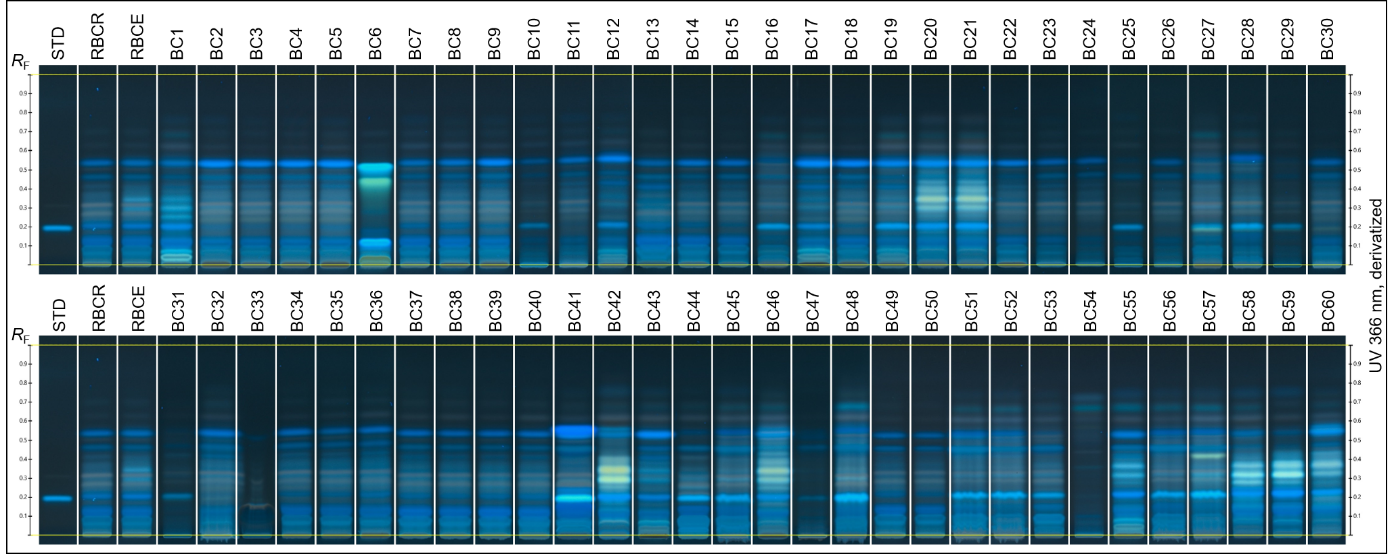


**Figure S6** HPTLC fingerprint of all black cohosh products (BCP) and ingredients (BCI) under UV 366 nm after derivatization with Antimony trichloride reagent. Test for A. dahurica. STD: cimifugin; RBCR: reference black cohosh root; 5% AD: A. racemosa mixed with 5% of A. dahurica.


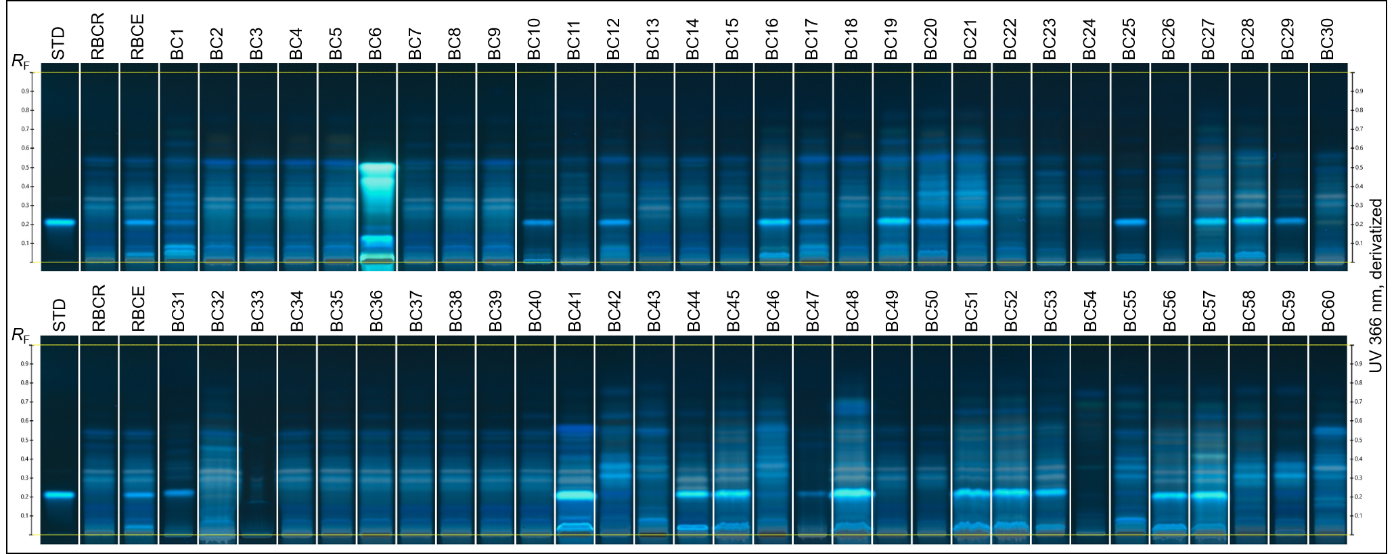


**Figure S7** HPTLC fingerprint of all black cohosh products (BCP) and ingredients (BCI) under UV 366 nm after derivatization boric acid/oxalic acid reagent. Test for A. cimicifuga. STD: cimifugin; RBCR: reference black cohosh root; 5% AD: A. racemosa mixed with 5% of A. cimicifuga.

# *Calculation of the costs for preparation and analysing the samples*

The prices per mL of high-purity solvents (used to prepare the mobile phase and samples), of the stationary phases and single use disposable material were obtained from Sigma Aldrich and Merck websites: https://www.sigmaaldrich.com/switzerland-suisse.html; http://www.merckmillipore.com/CH/de; (accessed on 20.12.2019 and 24.01.2020).

Calculation excludes standards, instruments, glass apparatus, and personal.

1. Preparation cost per sample: the prices per mL of solvent used in each method and single use disposable material such as centrifuge tubes, plastic pipettes, and syringe filter, were used for calculation. Echinacea products are prepared twice, each with a different method.
2. The costs for HPTLC analyses per sample were calculated based on:
   1. The volume of solvent used per plate.
   2. The price of a single plate
   3. The cost per derivatization, taking into account the price of solvents and reagents needed to prepare 200 mL. The obtained value is divided by 50 (approximated number of times a solution is used to dip a per plate).

Note: This calculation excluded the price per filter paper because it is not predictable how many times a single sheet can be used for the USP HPTLC method.
